# The Divergent CD8^+^ T Cell Adjuvant Properties of LT-IIb and LT-IIc, Two Type II Heat-Labile Enterotoxins, Are Conferred by Their Ganglioside-Binding B Subunits

**DOI:** 10.1371/journal.pone.0142942

**Published:** 2015-11-13

**Authors:** John C. Hu, Christopher J. Greene, Natalie D. King-Lyons, Terry D. Connell

**Affiliations:** Department of Microbiology & Immunology, The Witebsky Center for Microbial Pathogenesis and Immunology, The University at Buffalo, Buffalo, New York, United States of America; The Ohio State University, UNITED STATES

## Abstract

Poor immune responses elicited by vaccine antigens can be enhanced by the use of appropriate adjuvants. Type II heat-labile enterotoxins (HLT) produced by *Escherichia coli* are extremely potent adjuvants that augment both humoral and cellular immunity to co-administered antigens. Recent findings demonstrate that LT-IIb and LT-IIc, two type II HLT adjuvants, exhibit potent, yet distinguishable CD8^+^ T cell adjuvant properties. While LT-IIc elicits a robust and rapid response at one week after administration, LT-IIb engenders a more gradual and slower expansion of antigen-specific CD8^+^ T cells that correlates with improved immunity. The variations in immune effects elicited by the HLT adjuvants have been generally attributed to their highly divergent B subunits that mediate binding to various gangliosides on cell surfaces. Yet, HLT adjuvants with point mutations in the B subunit that significantly alter ganglioside binding retain similar adjuvant functions. Therefore, the contribution of the B subunits to adjuvanticity remains unclear. To investigate the influence of the B subunits on the enhancement of immune responses by LT-IIb and LT-IIc, chimeric HLT were engineered in which the B subunits of the two adjuvants were exchanged. Comparing the immune potentiating characteristics of both native and chimeric HLT adjuvants, it was found that not all the adjuvant characteristics of the HLT adjuvants were modulated by the respective B subunits. Specifically, the differences in the CD8^+^ T cell kinetics and protective responses elicited by LT-IIb and LT-IIc did indeed followed their respective B subunits. However, induction of IL-1 from macrophages and the capacity to intoxicate cells in a mouse Y1 adrenal cell bioassay did not correlate with the B subunits. Therefore, it is likely that additional factors other than the B subunits contribute to the effects elicited by the HLT adjuvants.

## Introduction

In combination with improved sanitation, vaccination remains a cornerstone in the fight against infectious diseases [1]. The global eradication of smallpox and the dramatic decrease in the prevalence of polio, measles, tetanus, diphtheria, and numerous other diseases validate the undeniable benefits of vaccines to human health. Yet, prospective vaccines to prevent or treat other serious pathogens, such as human immunodeficiency virus (HIV), hepatitis C virus (HCV), and herpes virus have not yielded true clinical successes. This failure is due, in part, to the complex nature of these pathogens and to the requirement for precise and appropriate immune responses to proffer both protection and clearance [2]. While the generation of neutralizing antibodies that prevent primary infection remains a fundamental goal of vaccine development, another critical line of defense is the elicitation of effector T cells, especially when high neutralizing titers are not attainable or are insufficient for complete protection [3–5]. Additionally, the development of therapeutic vaccines that can be deployed post-infection remains an important objective, especially in chronic infections such as HIV and HCV [6, 7]. In these cases, the sole reliance on antibody-mediated clearance is inadequate as intracellular pathogen populations are shielded from the humoral immune response. Therefore, the engagement of CD4^+^ T cells and CD8^+^ T cells that display critical effector functions such as antiviral cytokines or direct lytic capabilities is paramount for the effective clearance of such intracellular organism and disease prevention.

Recombinant protein antigens in vaccines typically do not induce a strong effector T cell response [8]. In those cases, appropriate adjuvants that can robustly enhance both the humoral and T cell responses are critical for development and application of novel vaccines that seek to stimulate cellular immunity. Heat-labile enterotoxins (HLT), which are among the most potent adjuvants that have been described, induce both humoral and cellular immunity [9–12]. The HLT superfamily of adjuvants is divided into two subgroups: type I and type II. The members of the two subgroups are distinguishable at the antigenic and genetic levels [13, 14]. Type I HLTs include cholera toxin and LT (referred herein as LT-I), produced by *Vibrio cholerae* and *Escherichia coli*, respectively. Both CT and LT-I have been examined exhaustively in terms of adjuvant properties [9, 13]. In contrast, the adjuvant properties of the type II HLT that includes LT-IIa, LT-IIb, and LT-IIc have only recently been explored.

Both type I and type II HLT adjuvants share structural similarities. Each HLT is composed of an A polypeptide that is non-covalently linked to a pentameric ring of B polypeptides [15]. The A polypeptide, or subunit, catalyzes the ADP-ribosylation of the intracellular Gsα regulatory protein [13]. Ribosylation of Gsα induces constitutive activation of the cell’s adenylate cyclase, thereby dramatically increasing the intracellular concentration of cAMP, a signaling molecule for a variety of cellular functions. The B subunits of the HLT adjuvants mediate binding to one or more types of gangliosides that are ubiquitously expressed on cell surfaces. Notably, the preferences for binding to gangliosides are determined by the divergence in the amino acid sequences of the B polypeptides of each of the types of HLTs [15, 16]. While it is evident that the type II HLTs are strong adjuvants, several fundamental questions in regards to the mechanisms that underlie the adjuvant properties of type II HLT have yet to be fully addressed.

One such question involves the role of the B pentamers in adjuvanticity. It has been proposed that the differences in the patterns of immune responses induced by the various HLT adjuvants are driven by the affinity of each HLT to its specific ganglioside receptors. Experimentally, however, the contribution of the B subunit to adjuvanticity is unclear. For example, LT-I, a type I HLT, binds the ganglioside GM1 with high affinity and has been shown to induce a protective CD8^+^ T cell response when delivered intradermally [17]. A glycine to aspartic acid substitution (G33D) in the B subunit of LT-I that completely abrogates GM1 binding, however, does not ablate that HLT’s CD8^+^ T cell adjuvant properties [17]. Furthermore, mutations to the B subunits of LT-IIa and LT-IIb that altered their receptor binding affinities did not interfere with those adjuvants’ capacities to augment antigen-specific humoral immune responses when co-administered with a poor immunogen by the intranasal route [10, 18]. Likewise, mutant LT-IIb(T13I), which has significantly reduced affinity for its ganglioside receptors [19], does not exhibit detectible decreases in adjuvant characteristics in comparison to wild-type LT-IIb when co-administered with a variety of antigens by the intranasal or the intradermal route [10, 20]. Therefore, the fundamental question of whether the B pentamers of HLTs convey specific adjuvant characteristics remains to be addressed.

While the type II HLT adjuvants LT-IIb and LT-IIc are potent antigen-specific CD8^+^ T cell adjuvants, each has distinguishable characteristics [12]. In a murine intradermal immunization model, LT-IIc induced a rapid expansion of antigen-specific CD8^+^ T cells, while the effects of LT-IIb were observed to have much slower kinetics, but with an improved long term efficacy. Critically, in comparison to the effects of LT-IIc and LT-I, clearance of *Listeria monocytogenes*, an intracellular pathogen, was more efficient upon challenge one month after a single immunization when LT-IIb was employed as the intradermal adjuvant [12]. By taking advantage of the unique observation that LT-IIb and LT-IIc induces definable and different CD8^+^ T cell enhancements, we utilized genetic techniques to determine if the different B subunits convey the immune response exhibited by these two HLT adjuvants. Two chimeric HLT adjuvants were engineered by exchanging the B subunits to produce two new HLT adjuvants: LT-IIcb (LT-IIc A subunit with the LT-IIb B pentamer) and LT-IIbc (LT-IIb A subunit with the LT-IIc B pentamer). Comparing LT-IIcb and LT-IIbc to their wild-type (WT) counterparts revealed that the distinguishable CD8^+^ T cell immune responses induced by the HLTs were harbored within their respective B pentamers. However, we found that other immunomodulatory effects of the HLTs were not conferred by the B subunits. Specifically, the capacity to induce IL-1 from macrophages by the chimeric HLT adjuvants, however, did not follow that of the originating B subunits. Thus, the unique and contrasting immune effects induced by LT-IIb and LT-IIc and with the novel chimeric HLT adjuvants provide an excellent platform to better understand and dissect the intricacies, at a fundamental level, by which these potent adjuvants augment Ag-specific immune responses.

## Methods

### Mice and immunizations

Female 8–12 week C57Bl/6J mice (Jackson Laboratory, Bar Harbor, ME) were utilized for all immunization experiments. Mice were anesthetized with ketamine (75 mg/kg) and xylazine (10 mg/kg) and the lower back was shaved with electric clippers. The administration site was sterilized with 70% isopropanol and vaccine preparation was injected ID using a 29G needle and 0.5 cc insulin syringe (BD, Franklin Lakes, NJ) with the bevel side up. Correct ID administration was visually confirmed as a small induration with clear margins after injection. Vaccine preparation consisted of chicken egg ovalbumin (OVA) (Sigma, St. Louis, MO) at 50 μg with and without 1 μg of HLT adjuvant in 30 μl of phosphate buffered saline (PBS). Mice were euthanized by CO_2_ asphyxiation and/or cervical dislocation at the termination of the experiments. All experiments employing animals were approved by the Institutional Animal Care and Use Committee (IACUC) at The University at Buffalo (Approval #MIC01010Y).

### Construction of chimeric HLT adjuvants

Chimeric HLT adjuvant constructs were engineered using overlap extension polymerase chain reactions [21]. Briefly, plasmid pHN1 for LT-IIb [14] and pJCH6.2 for LT-IIc [22] were utilized to amplify A and B subunits having overlapping primer regions. This initial amplification was followed by a second PCR step to promote overlap extension (Table 1). Spliced constructs were created by a second PCR step which incorporated LT-IIa A subunit and LT-IIc B subunit fragments and 5’ and 3’ terminal primers. Easy A polymerase (Agilent, Santa Clara, CA) was employed for the second PCR step. Chimeric PCR products were cloned into pGEM-T plasmids (Promega, Madison, WI) and transformed into *E*. *coli* DH5αF’Kan. These plasmids were digested with *Bam*HI and *Xba*I, and the fragments subcloned into pBluescript KS+ for subsequent transformation into *E*. *coli* DH5αF’Kan for protein expression.

**Table 1:** Primers and products for construction of chimeric HLT adjuvants.

Template	Primers	Product
LT-IIb plasmid	5-GGATCCATGCTCAGGTGAGAAT-3	LT-IIb A subunit
	5-AATTGACTTTTTAAAGCTCATCTTTTCCTCCATTA-3	
LT-IIb plasmid	5-TAATGGAGGAAAAGATGAGCTTTAAAAAGTCAATT-3	LT-IIb B subunit
	5-TCTAGATTAGTGGTGGTGGTGGTGGTGTTCTGCCT-3	
LT-IIc plasmid	5-GGATCCCTGGAGCTGGCAGAGA-3	LT-IIc A subunit
	5-GATAATTTTCTTAAAGCTCATCTTTATTTTCATTG-3	
LT-IIc plasmid	5-CAATGAAAATAAAGATGAGCTTTAAGAAAATTATC-3	LT-IIc B subunit
	5-TCTAGATTAGTGGTGGTGGTGGTGGTGTGGTGCTAA-3	
**Overlap Extension**		
LT-IIb A subunit	5-GGATCCATGCTCAGGTGAGAAT-3	LT-IIbc chimera
LT-IIc B subunit	5-TCTAGATTAGTGGTGGTGGTGGTGGTGTGGTGCTAA-3	
LT-IIc A subunit	5-GGATCCCTGGAGCTGGCAGAGA-3	LT-IIcb chimera
LT-IIb B subunit	5-TCTAGATTAGTGGTGGTGGTGGTGGTGTTCTGCCT-3	

### Antigen, adjuvant preparation, and analysis

Recombinant HLT adjuvants were purified by nickel affinity and gel chromatography [14]. Protein concentration was determined by Micro BCA Protein Assay Kit (Pierce, Rockford, IL). LPS contamination was determined by the use of a Limulus Amoebocyte Lysate Endochrome Kit (Charles River Endosafe, Charleston, SC). All purified protein preparations had ≤ 0.03 ng of LPS per μg of protein. Protein adjuvants were analyzed by SDS-PAGE and Coomassie blue staining [22]. Nicking of the A polypeptide was performed by incubating 20 μg of HLT for 4 hr at 37°C with 10 μg/mL of trypsin (Sigma) dissolved in 10 mM HCl.

### Ganglioside binding blots

Commercially available gangliosides GM1, GM2, GM3, GD1a, and GD1b (Matreya, State College, PA) were diluted to 500 μm/μL in methanol. One μL of dissolved ganglioside was dotted onto low fluorescence polyvinylidene fluoride membranes (Life Technologies, Grand Island, NY) in a grid pattern. Membranes were dried at RT for 30 min and blocked with Blocking Buffer (PBS, 2% BSA) for 1 hr at RT. Gangliosides were incubated with 2 μg/mL HLT in Blocking Buffer for 1 hr at RT and washed with PBS. Blots were probed with anti-6x-HIS antibody (1:2000, Clone HIS.H8, Thermo Fisher Scientific, Rockford IL) for 1 hr followed by goat anti-mouse IgG AF680 (1:8000, Life Technologies, Grand Island NY), all antibodies diluted in Blocking Buffer with 3x PBS washes between incubations. Blots were analyzed at 700 nm using a LiCOR Odyssey CLx (Lincoln, Nebraska).

### Toxicity bioassay

Mouse Y1 adrenal cells (ATCC #CCL-79) were cultured to 70–85% confluence in 96-well plates in RPMI 1640 containing 10% FCS, 100U of penicillin, and 100U of streptomycin. Cells were exposed to HLT adjuvants starting at 1 μg/well followed by 11 two-fold dilutions. Four hours after treatment, cells were examined for rounding as an indicator of intoxication. The concentration at which 75–100% of the cells exhibiting rounding was considered as 1 unit of activity.

### Stimulation of peritoneal macrophages

Peritoneal macrophages were isolated from C57BL/6 mice [23]. Macrophages were treated with 1 μg/mL of LPS for 4 hr prior to treating overnight with 2 μg/mL of LT-IIb, LT-IIc, LT-IIbc, or LT-IIcb. Culture supernatants were collected the following day and stored at -80°C until analyzed by ELISA. Treated macrophages were detached from the cell culture wells with enzyme-free EDTA disassociation buffer (Sigma) and mechanically disrupted with cell scrapers (Corning, Tewksbury MA). Cells were filtered through 40 μm nylon cell strainer (Corning) prior to staining for flow cytometry.

### Flow cytometry and antibodies

Peripheral blood cells were collected from the facial vein into microfuge tubes containing 10 μL of 0.5M EDTA. Erythrocyte were lysed using RBC lysis buffer (eBioscience, San Diego, CA). Cells were washed in FACS buffer (PBS, 0.1% heat-inactivated fetal calf serum, 0.01% sodium azide), stained for extracellular markers, and fixed in 4% paraformaldehyde. OVA-specific dextramer was purchased from Immudex (Copenhagen, Denmark). Aqua LD stain (Life Technologies, Grand Island, NY) was employed for live/dead differentiation. Antibodies were obtained from eBioscience, Biolegend (San Diego, CA), or BD Biosciences (San Jose, CA): TCRβ (Clone H57-597), CD8α (Clone 53–6.7), CD44 (Clone IM7), CD11b (Clone M1/70), and pro-IL-1b (Clone NJTEN3), and CD86 (Clone GL1). FCR blocking was performed using anti-CD16/32 (Clone 93, Ebioscience). Data was captured using an LSR-Fortessa (BD Biosciences) and analyzed using FlowJo software (Treestar, Ashland, OR).

### Cytokine ELISA

Culture supernatant from treated peritoneal macrophages were assayed by ELISA for IL-1β (Biolegend) and IL-1α (eBioscience).

### *Listeria monocytogenes* challenge

Recombinant *L*. *monocytogenes* (rLM-OVA) expressing OVA134–387 (strain DMX 09–082) was purchased from DMX Inc. (Philadelphia, PA). rLM-OVA was cultured in Brain Heart Infusion (BHI) broth overnight at 37°C with agitation. Bacteria were pelleted by centrifugation and washed twice, resuspended in PBS, and frozen at −80°C. CFU of the frozen stocks were determined after thawing. For non-lethal challenges, immunized mice were injected with one LD_50_ (5×10^6^ CFU) i.v. [17] and euthanized after 3 days to enumerate CFU of rLM-OVA in the spleen. Serial dilutions of spleen homogenates were inoculated onto BHI agar plates containing 100 μg/mL erythromycin and the plates incubated at 37°C for two days prior to colony counting. For lethality and protections studies, mice were inoculated with 10 LD_50_ (5×10^7^ CFU) i.v. and observed each day for 7 days. Moribund mice were euthanized and recorded as lethal experimental endpoints.

### Statistical analysis

All data were analyzed using Graphpad Prism (GraphPad Software, Inc., La Jolla, CA).

## Results

### B subunits of LT-IIb and LT-IIc determine ganglioside binding and holotoxin stability

**Fig 1:**
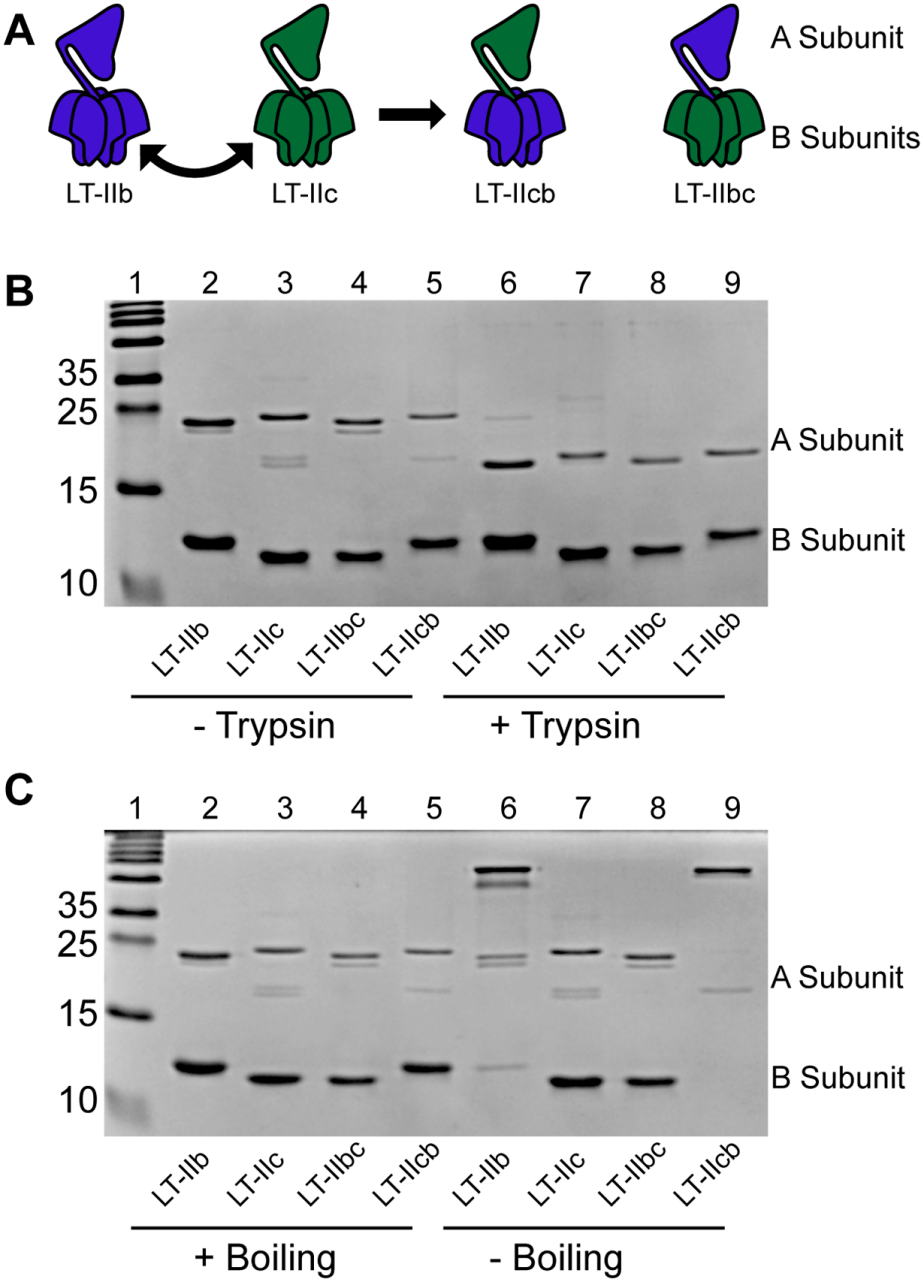
Construction and purification of chimeric HLT adjuvants. (A) Schematic of swapping the B subunits of LT-IIb and LT-IIc to generate new chimeric HLT adjuvants LT-IIcb (LT-IIc A subunit + LT-IIc B subunits) and LT-IIbc (LT-IIb A subunit + LT-IIc B subunits). (B) 15% SDS-PAGE gel stained with Coomassie Brilliant Blue of uncleaved (lanes 2–5) and trypsin cleaved (lanes 6–9) WT and chimeric HLTs. (C) 15% SDS-PAGE gel stained with Coomassie Brilliant Blue of samples boiled (lanes 2–5) or unboiled (lanes 6–9) in SDS sample buffer prior to electrophoresis.

To better understand the role of the A and B subunits of LT-IIb and LT-IIc in the distinct CD8^+^ T cell adjuvant effects, chimeric adjuvants were constructed by genetically exchanging the B pentamers of the two HLTs (Fig 1A). The two novel chimeric HLTs were purified and designated LT-IIcb and LT-IIbc, where the first letter indicated the origin of the A subunit and the second letter indicating the origin of the B pentamer. Correct functioning of WT and the chimeric HLT holotoxins requires proper proteolytic cleavage and reduction of a disulfide bond between the A1 and the A2 domains that releases the A1 subunit from the holotoxin after internalization [24]. To ensure protease accessibility of the A subunit, exogenous trypsin and 2-mercaptoethanol (2-ME) was used to evaluate the capacity of the chimeric HLT adjuvants to be properly cleaved and the subunits to be separated *in vitro*. Both chimeric HLT adjuvants were cleavable by trypsin and reducible by 2-ME to yield the expected smaller A1 polypeptides (Fig 1B).

Additionally, both LT-IIb and LT-IIcb remained in native holotoxin forms when resolved by SDS-PAGE in the absence of boiling. Conversely, both LT-IIc and LT-IIbc separated into A and B subunits in the absence of heating in SDS loading buffer prior to resolution by SDS-PAGE (Fig 1C). This difference suggested that the B subunits of LT-IIb in both wild-type LT-IIb and chimeric LT-IIcb conferred better stability to the holotoxins, independent of the originating A subunit, than did the B subunits of either LT-IIc or LT-Ibc, which exhibited less stability in SDS.

**Fig 2:**
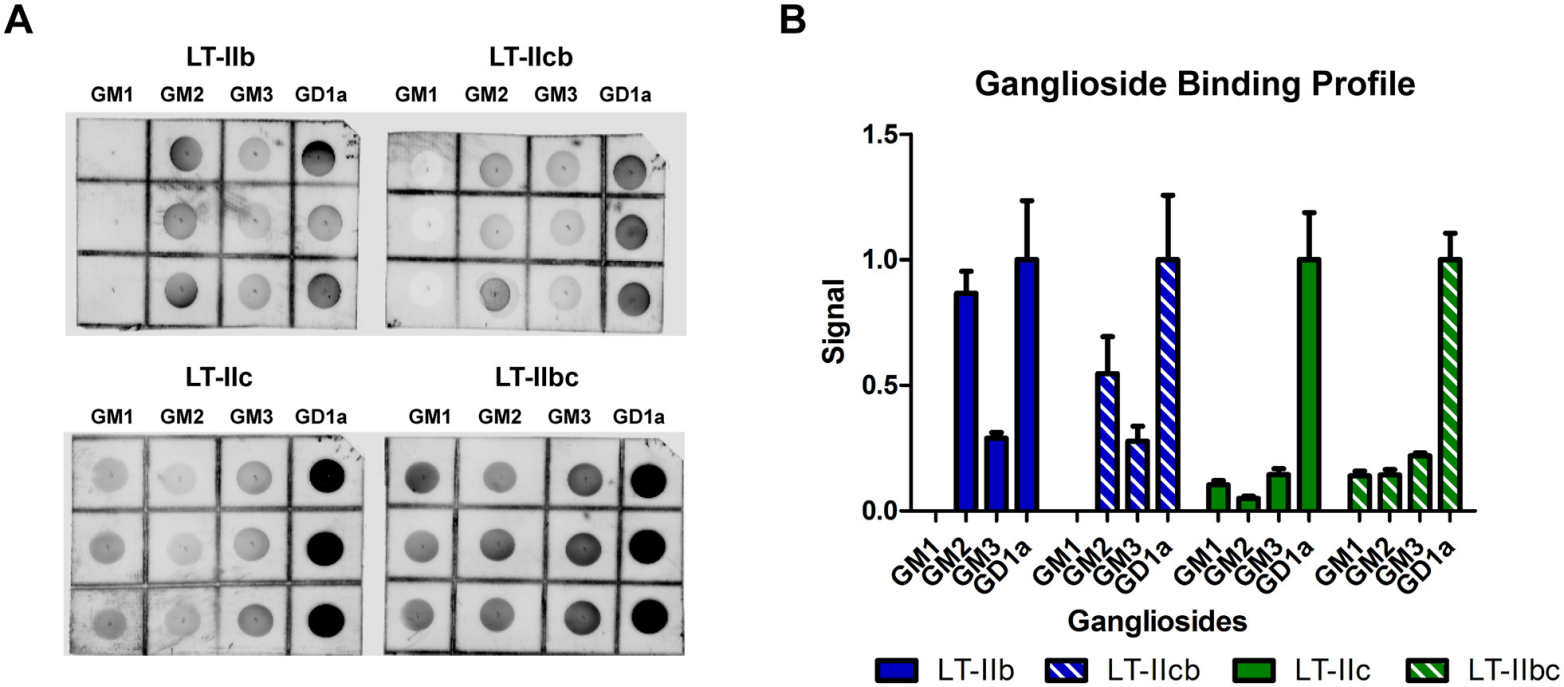
Chimeric HLT adjuvants preserve parental B-pentamer ganglioside binding profiles. (A) Modified dot blots with 500 μm of each ganglioside spotted on a grid and probed with 2 μg/mL HLT adjuvant, anti-HLT polyclonal antibodies, and anti-rabbit IgG AF790. (B) Binding of both WT and chimeric HLTs to different gangliosides as normalized to GD1a binding. Data shown (n = 3).

The pentameric B subunits of the HLT adjuvants are responsible for binding gangliosides expressed ubiquitously on cell surfaces. To ensure that the chimeric HLT adjuvants preserved their parental ganglioside binding profiles, modified immunodot blots using commercially prepared gangliosides were performed (Fig 2A). As previously reported, LT-IIb binds GM2, GM3, and GD1a, but not GM1, while LT-IIc binds all four of the mentioned gangliosides [22]. Binding to particular gangliosides was dependent upon the B pentamer that was present in the chimeric holotoxins and equivalent to the WT HLT from which the B pentamers were derived. LT-IIcb bound to GM2, GM3, and GD1a, but not to GM1 (Fig 2B). Similarly, LT-IIbc bound to all four gangliosides: GM1, GM2, GM3, and GD1a (Fig 2B).

Thus, the B subunits of LT-IIb and LT-IIc not only dictate the ganglioside binding profiles of each HLT adjuvant, but also contribute to the overall stability of the holotoxin.

### Cytotoxicity of WT and chimeric HLT adjuvants

To compare the novel chimeric adjuvants to their WT counterparts, the WT and chimeric holotoxins were analyzed for toxicity using a mouse Y1 adrenal cell bioassay, the gold standard for assessing cytotoxicity of HLTs [23, 25]. Y1 adrenal cells are exquisitely sensitive to cAMP intoxication and respond by morphologically changing from flattened to rounded cells visible by microscopy. Careful titration of very low concentration of HLTs allow for a relative comparison and indirect quantification of cAMP activity and cytotoxicity. Utilizing this assay, and in agreement with previously published data, we found that LT-IIc was half as cytotoxic as LT-IIb [22] (Table 2). Curiously, however, cytotoxicity exhibited by the chimeric adjuvants did not completely mirror the cytotoxicity of the parental HLTs from which their B pentamers were derived. LT-IIcb was 8-fold more cytotoxic than LT-IIb, with which it shares the B pentamer; LT-IIbc was 5-fold less toxic in comparison to LT-IIc (Table 2). Just as LT-IIb is more cytotoxic to Y1 adrenal cells than LT-IIc, LT-IIcb is also more cytotoxic when compared to LT-IIbc. These differences, however, were greatly accentuated in the chimeric enterotoxins, as LT-IIb displayed only twice the toxicity of LT-IIc, while LT-IIcb was 80-fold more toxic than LT-IIbc.

**Table 2:** Cytotoxicity of wild-type and chimeric HLT adjuvants.

HLT Adjuvant	Toxicity Concentration^a^	Relative Units of Toxicity^b^
LT-IIb	15.63	1.0
LT-IIcb	1.95	8.0
LT-IIc	31.25	0.5
LT-IIbc	250.00	0.1

^a^ Concentration of HLT(ng)/well to induce 75–100% of cell rounding

^b^ Reciprocal of the toxicity concentration relative to LT-IIb

### Induction of IL-1 in macrophages

**Fig 3:**
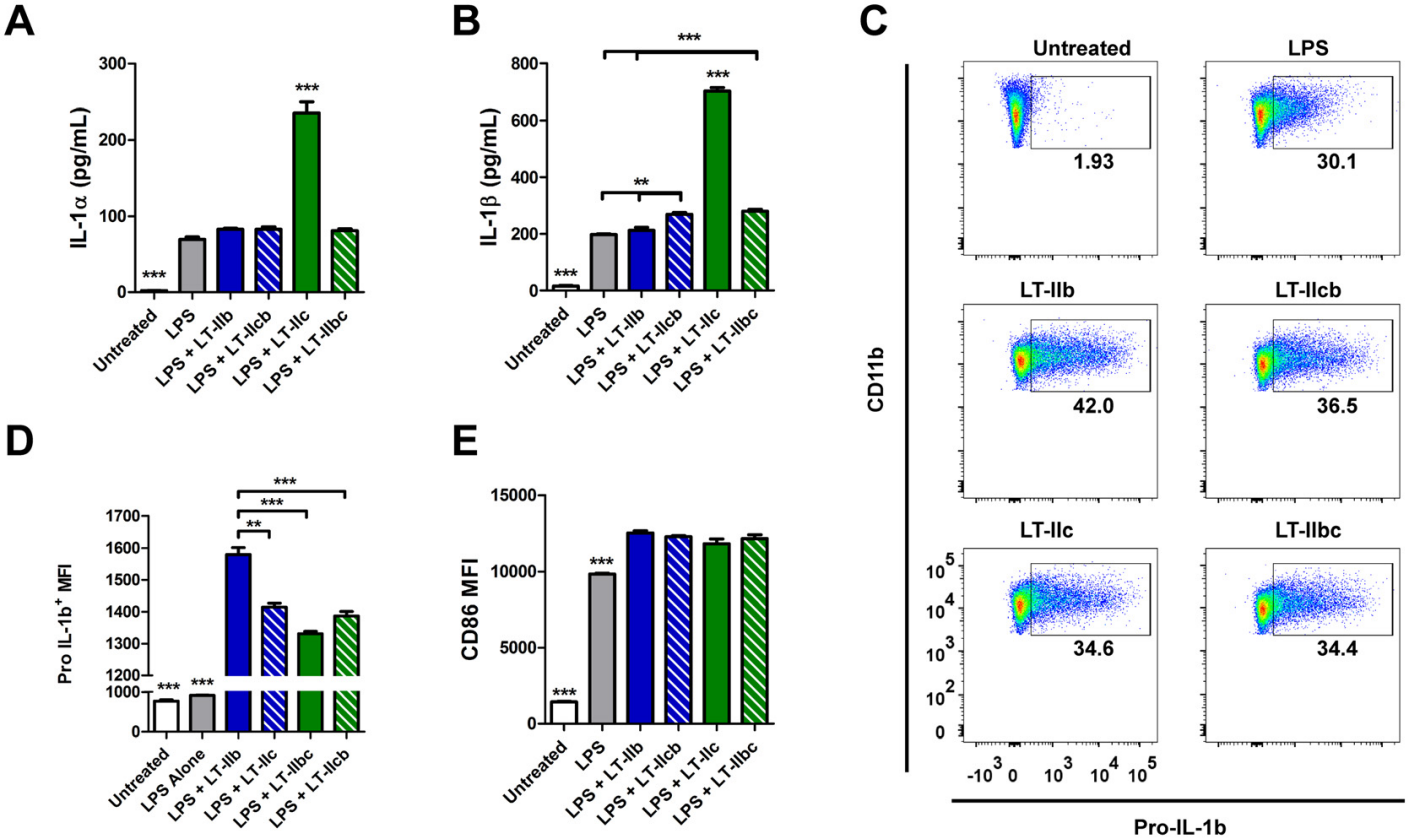
Enhancement of IL-1 production by WT and chimeric HLT adjuvants. Thioglycollate-induced peritoneal macrophages were treated overnight with LPS and HLT adjuvants *in vitro*. (A) Concentration of IL-1α in the culture supernatant after overnight treatment. (B) Concentration of IL-1β in the culture supernatant after overnight treatment. (C) Staining of uncleaved pro-IL-1β in peritoneal macrophages after overnight treatment. (D) Expression of pro-IL-1β by peritoneal macrophages after *in vitro* treatment by WT and chimeric HLT adjuvants. (E) Mean fluorescence intensity (MFI) of CD86 as a marker for activation in treated peritoneal macrophages. Data shown (n = 3). *Statistical Analysis*: One-way ANOVA with Bonferroni post-test. *P ≤ 0.05; **P ≤ 0.01; ***P ≤ 0.001 compared to all other groups unless indicated.

LT-IIc is the newest member of the type II HLT family [9, 12, 22, 23]. In comparison to all other type II HLTs, LT-IIc was found to profoundly enhance the production of both IL-1α and IL-1β in LPS-stimulated peritoneal macrophages [23]. To determine if the new chimeric HLT adjuvants, and specifically LT-IIbc, maintained this unique property, the effects of both WT and chimeric HLT on peritoneal macrophages were evaluated. Analogous with the previous report, LT-IIc potentiated significantly more IL-1α and IL-1β secretion in LPS-primed peritoneal macrophages in comparison to LT-IIb and controls (Fig 3A and 3B). Surprisingly, the effects of both LT-IIbc and LT-IIcb on macrophages were similar to those elicited by LT-IIb; neither chimeric HLT elevated secretion of IL-1α to levels equivalent to those elevated by LT-IIc (Fig 3A and 3B). Processing of pro-IL-1β into the mature IL-1β is required prior to release from the cell. Therefore, intracellular levels of pro-IL-1β were examined in these macrophages (Fig 3C). While all HLT treated macrophages had higher levels of intracellular pro-IL-1β than did macrophages treated only with LPS or mock treated controls, levels of intracellular pro-IL-1β were highest in cells treated with LT-IIb (Fig 3C and 3D). Additionally, we found that LT-IIb treated cells also exhibited higher percentages of macrophages that expressed pro-IL-1β (Fig 3C, S1 Fig) when compared to the other groups. This observation was surprising as mature, secreted IL-1β was not significantly elevated in the culture supernatant of LT-IIb-treated macrophages in comparison to the other HLT-treated groups. This result suggested that LT-IIb influences processing and/or expression of pro-IL-1β, thus causing an accumulation of the uncleaved form of the cytokine, a property unique to LT-IIb. Finally, all HLT-treated macrophages exhibited a more activated and mature phenotype when compared to macrophages treated solely with LPS, as measured by the expression of CD86 (Fig 3E). CD86 expression was equivalent among the HLT treated groups.

Therefore, both LT-IIb and LT-IIc exhibited unique effects of peritoneal macrophages that are otherwise not exhibited by the chimeric HLTs and suggests that the B subunits are not solely responsible for all of the immune effects induced by LT-IIb and LT-IIc.

### B pentamers convey CD8^+^ T cell adjuvant characteristics

**Fig 4:**
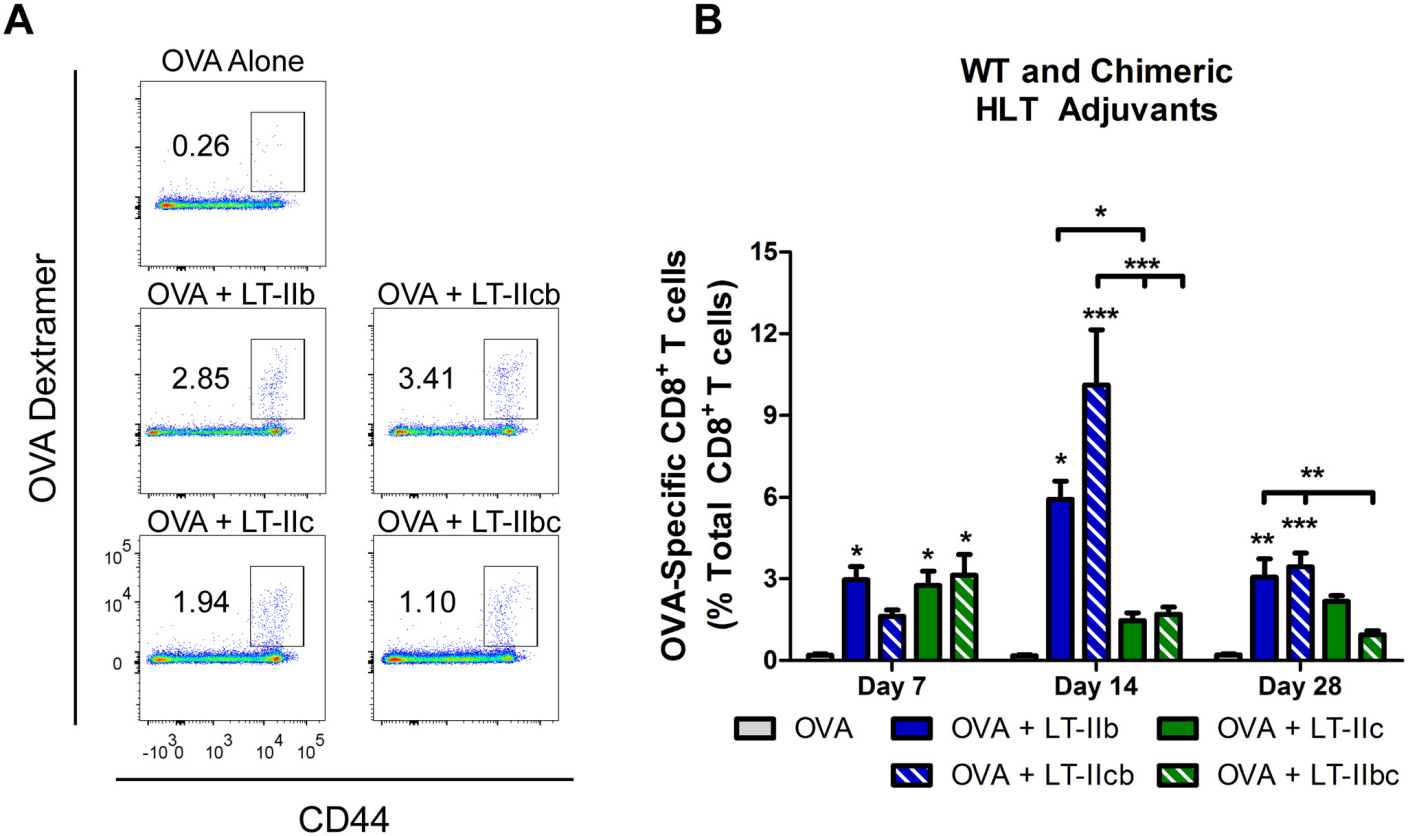
CD8+ T cell responses to chimeric HLT adjuvants. OVA-specific CD8^+^ T cells in the peripheral blood post-immunization utilizing 50 μg of OVA and 1.0 μg of WT or chimeric HLT adjuvants. (A) Staining of OVA-specific CD8^+^ T cells from peripheral blood 28 days after immunization. Cells gated from live TCRβ^+^CD8^+^ population. (B) OVA-specific CD8+ T cells from PBMCs after 7, 14, and 28 days post-immunization. Data shown (n = 8). *Statistical analysis*: One-way ANOVA with Bonferroni post-test compared to OVA, unless otherwise noted. *P ≤ 0.05; **P ≤ 0.01; ***P ≤ 0.001. Results shown as the arithmetic mean with error bars denoting SEM.

The immunomodulatory differences exhibited by the various HLTs is hypothesized to be determined by the highly divergent B subunits that target the adjuvant to different immune cells or cellular compartments due to the HLTs’ different ganglioside preferences [13]. While both LT-IIb and LT-IIc enhance an antigen-specific CD8^+^ T response, LT-IIb induces a slower and longer expansion phase and promotes better vaccine efficacy upon pathogen challenge [12]. Therefore, to determine the contribution of the A and B subunits of LT-IIb and LT-IIc to these CD8^+^ T cell adjuvant characteristics, the responses elicited by both WT and chimeric HLT adjuvants in an intradermal immunization model were investigated. Mice were immunized with 1 μg of HLT adjuvant and 50 μg of ovalbumin (OVA), a model antigen. OVA-specific CD8^+^ T cells in the peripheral blood, identified by use of OVA dextramer staining (Fig 4A), were analyzed on days 7, 14, and 28 post-immunization. LT-IIbc exhibited CD8^+^ T cell expansion and contraction kinetics that were similar to those of wild-type LT-IIc with an early peak at day 7 followed by contraction for the duration of the experiment (Fig 4B). In contrast, the kinetics of the CD8^+^ T cell response was similar in cells treated with either LT-IIcb or WT LT-IIb, with a gradual expansion and contraction of OVA-specific CD8^+^ T cells (Fig 4B). Full expansion was not attained until 2 weeks after immunization. Amplitude differences, however, were observed when the responses to administration of LT-IIb and LT-IIcb were compared (Fig 4B).

**Fig 5:**
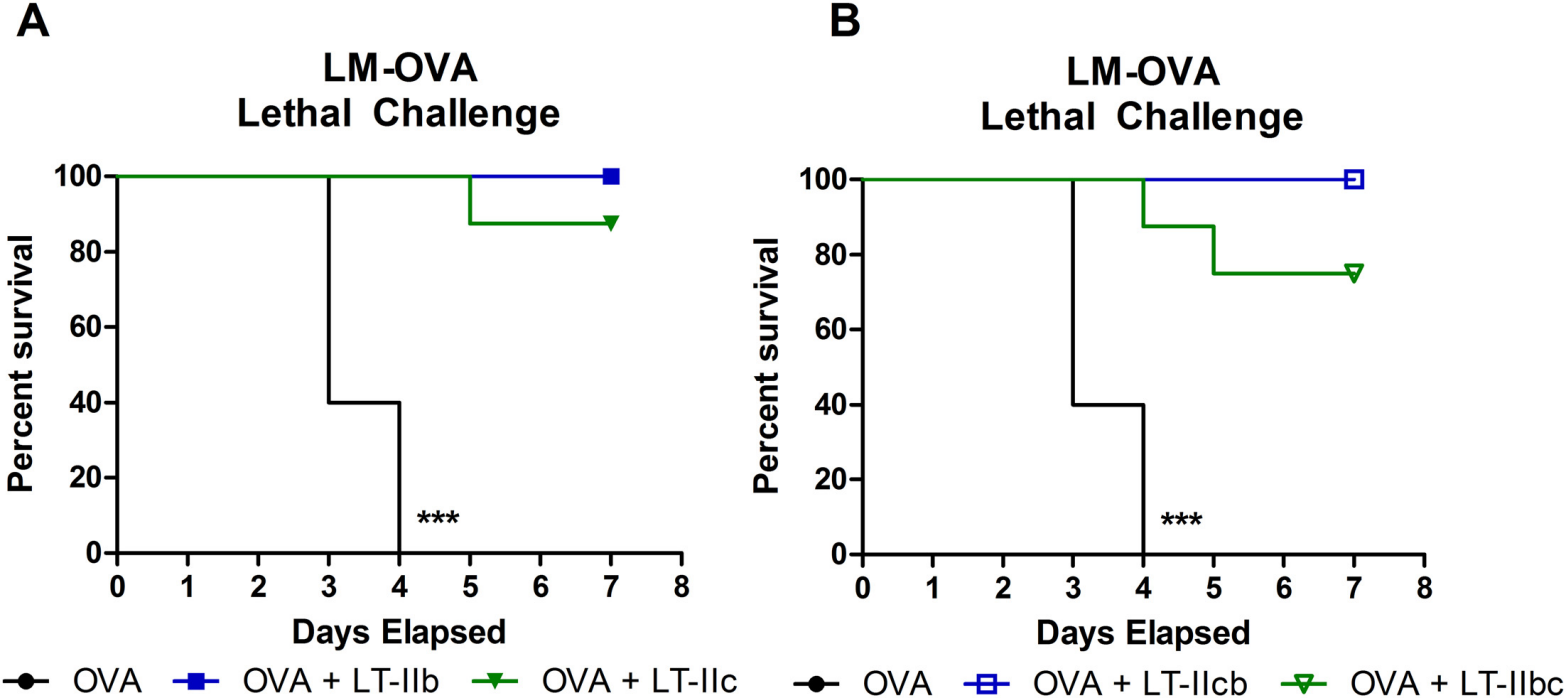
Protection against lethal rLM-OVA challenge. Kaplan-Meier plot of survival after lethal challenge with rLM-OVA (10xLD_50_, 5x10^7^ CFUs) 30 days after immunization with mice adjuvant with (A) WT or (B) chimeric HLT. Data shown (n = 8). *Statistical analysis*: Kaplan-Meier plot with Mantel-Cox Log-rank test. ***P ≤ 0.001.

To evaluate the protective response induced by both WT and chimeric HLTs, a lethal challenge with 10xLD_50_ (5x10^7^ CFU) of rLM-OVA was administered at a point 30 days after immunization. All mice that had received OVA alone succumbed to the infection by day 4 after inoculation. In contrast, HLT-adjuvanted mice exhibited a definite protective response (Fig 5). Groups adjuvanted with LT-IIb or LT-IIcb had 100% survival when lethally challenged with rLM-OVA. Most of the mice that had received LT-IIc or LT-IIbc as adjuvants survived the challenge, but protection was not comprehensive as one mouse died in the LT-IIc adjuvant group and two mice died in the LT-IIbc group (Fig 5). All surviving mice in the HLT adjuvant groups, both WT and chimeric, had complete clearance of detectable splenic rLM-OVA CFUs upon the end of the experiment at 7 days post-infection (data not shown).

These results indicated that both the CD8^+^ T cell kinetics and immune protection elicited by the chimeric HLT adjuvants mirrored that of the HLTs from which the B pentamers were derived. This survival data of the WT LT-IIb and LT-IIc correlates with the previously reported clearance data upon non-lethal challenge of rLM-OVA at 30 days post immunization showing that groups that received LT-IIb as an adjuvant evinced faster clearance rates when compared to the clearance rates in mice that had received the other HLT adjuvants [12]. Taken together, the data support the model that the highly divergent B subunits of LT-IIb and LT-IIc do, indeed, confer the distinctive CD8^+^ T cell enhancements elicited by these potent adjuvants.

### Functional A subunit of LT-IIb is critical for CD8^+^ T cell adjuvanticity

The role of the A subunit in the adjuvant effects of the HTL adjuvants is still under considerable debate [18]. In our model, LT-IIb induced a more protective CD8^+^ T cell response when compared to LT-IIc in our immunization-challenge model and showed clear differences in expansion and contraction kinetics (Figs 4 and 5). To elucidate the contribution of the ADP-ribosylation activity of the A subunit in the CD8^+^ T cell enhancements, the effects of WT LT-IIb were compared to those of LT-IIb(S59K, E108K). LT-IIb(S59K,E108K) contains two lysine substitutions within the A subunit that ablates the ribosylation activity of the adjuvant [26]. Using an ID immunization model, mice were administered either WT LT-IIb or LT-IIb(S59K, E108K) in the presence of OVA. OVA-dextramer was employed to track the kinetics of expansion and contraction of Ag-specific CD8^+^ T cells in the PBMC compartment.

**Fig 6:**
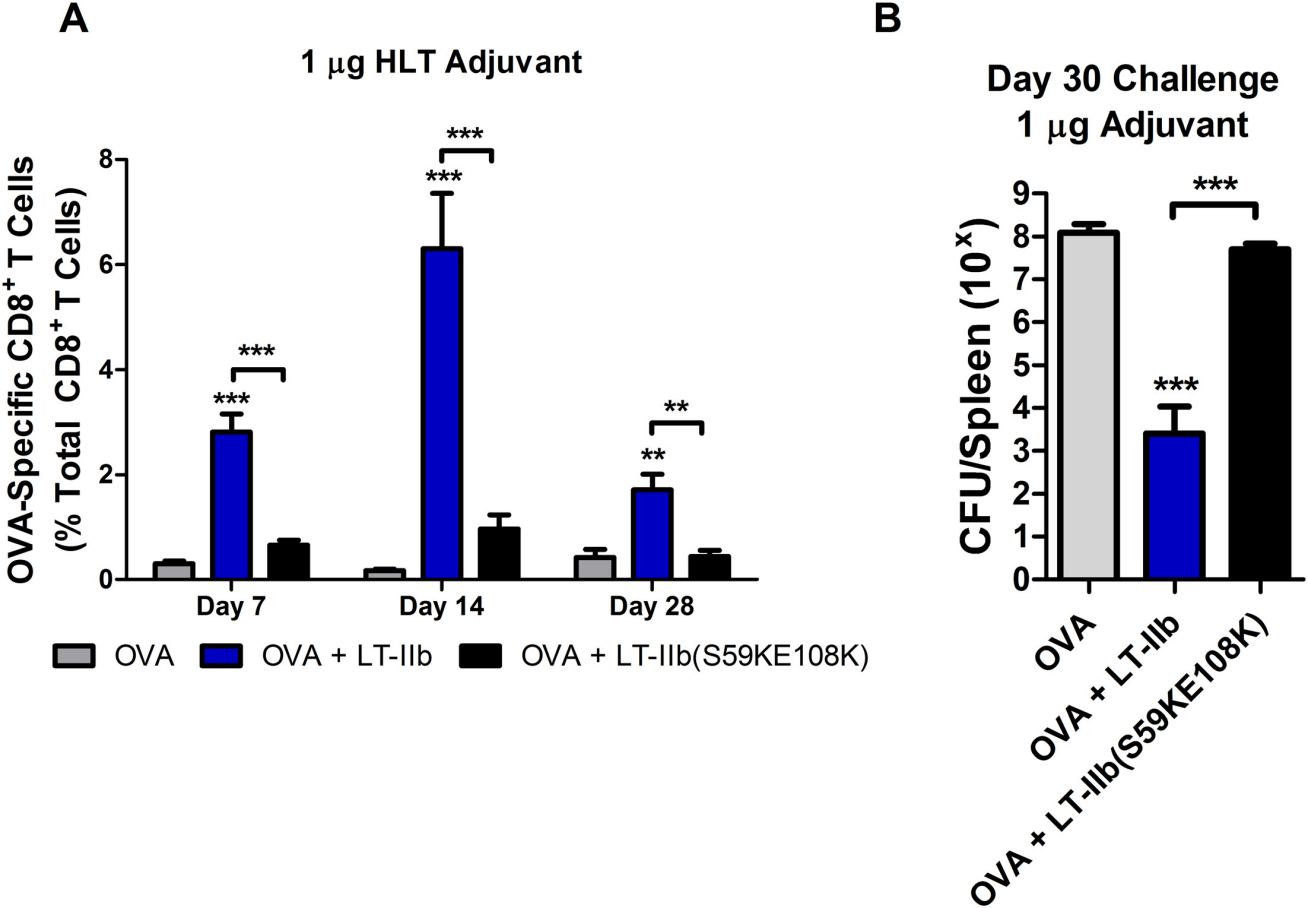
LT-IIb requires a functional catalytic domain for CD8^+^ T cell adjuvanticity. (A) OVA-specific CD8^+^ T cells from PBMCs after 7, 14, and 28 days post immunization utilizing 50 μg of OVA and 1 μg of LT-IIb or catalytic mutant LT-IIb(S56K, E108K). (B) Mice challenged with 1xLD50 (5x10^6^ CFU) of rLM-OVA 30 days post immunization and CFU enumerated from homogenized spleens after 3 days. Data shown (n = 8). *Statistical analysis*: One-way ANOVA with Bonferroni post-test compared to OVA alone unless otherwise noted. **P ≤ 0.01; ***P ≤ 0.001. Results shown as the (A) arithmetic or (B) geometric means with error bars denoting SEM.

LT-IIb(S59K, E108K) did not induce a statistically significant increase in circulating Ag-specific CD8^+^ T cells at any of the time points that were examined (Fig 6A). Subsequently, immunized mice were challenged 30 days after the immunization with a nonlethal load of rLM-OVA (5x10^6^ CFU). After three days, the mice were euthanized and the CFU of rLM-OVA were enumerated from the spleens. Once again, clearance rates were faster in mice that had received LT-IIb in comparison to mice that had received only OVA, with a ~5-log reduction in splenic CFU (Fig 6B). The enzymatically-deficient mutant LT-IIb(S59K, E108K) failed to induce better clearance of rLM-OVA than mice that had received OVA in the absence of adjuvant. These data suggests that the robust CD8^+^ T cell immunity induced by LT-IIb was dependent upon a fully enzymatically active A subunit.

## Discussion

The first vaccines developed with attenuated or inactive whole organisms contained natural danger signals that acted as adjuvants, a property largely taken for granted. Currently, the propensity to use well-defined subunit antigens in modern vaccine development often elicits sub-therapeutic responses during preclinical and clinical studies. The future success of vaccine strategies to target complex diseases and pathogens such as HIV, hepatitis, malaria, and malignancies will require a comprehensive engagement of the immune system including both robust neutralizing antibodies as well as effector T cells. Consequently, the understanding and expansion of the current repertoire of adjuvants is critical for the continuation of the vaccine enterprise. The capacity of the type II HLT adjuvants to enhance the humoral response in both magnitude and quality of neutralizing antibodies has been established [9, 10, 13, 14]. The capabilities of HLTs to augment cellular immunity have not been extensively evaluated. In that regard, the capacity of the type II HLT adjuvants to enhance CD8^+^ T cells [12] elevates their status as exceptional adjuvants with great clinical potential. Furthermore, the critical observation that LT-IIb and LT-IIc elicited different CD8^+^ T cell enhancements facilitated the ability to experimentally address some fundamental questions on the mechanics of these potent adjuvants. By constructing chimeric HLT adjuvants from LT-IIb and LT-IIc, we formally demonstrated that the different B subunits of the HLT adjuvants do, indeed, confer specific enhancements with tangible *in vivo* relevancy. Specifically, we found that HLT adjuvants, WT or chimeric, which contain the B subunit of LT-IIb, had similar delayed kinetics for CD8^+^ T cell expansion (Fig 4) and conferred better vaccine protection at a point one month after immunization in comparison to the effects of HLT adjuvants that contained the B subunit of LT-IIc (Fig 5).

It was also interesting to note that the B subunit of LT-IIb potentially enhanced overall holotoxin stability (Fig 1C). As the A and B subunits of all HLTs interact in a non-covalent manner, it could be expected that a WT holotoxin would exhibit the optimal configuration and interactions, as selected evolutionarily, for maximum environmental stability. However, in our assays, the B subunits of LT-IIb and LT-IIc were not optimally matched for stability; the B pentamer of LT-IIb was more stable in conjunction with the A subunit of either itself or with the A subunit of LT-IIc. Fortunately, the crystal structure of LT-IIb is known and can be used to predict the structure of LT-IIc for comparison. For all HLTs, the enzymatically active A subunit is non-covalently inserted into a pore formed by the B subunits arranged in a pentameric ring [15]. Utilizing alignment strategies and the known structure of LT-IIb, it can be noted that the pore region that is formed by the B subunits, and specifically the residues that form the solvent accessible component, is indeed different between LT-IIb and LT-IIc at two specific locations. Within this pore, LT-IIb contains two hydrophobic residues, valine 59 and alanine 62, that are instead hydrophilic in LT-IIc (tyrosine and asparagine, respectively). Therefore, it is feasible that the hydrophobicity of these two residues contributes to stabilizing A and B subunit interactions. In addition, the interactions between the B subunits could also be playing a fundamental role in the overall stability of the holotoxin. Thus, additional experiments are warranted to determine the means by which the intramolecular forces affect holotoxin stability and the overall implication of stability on HLT adjuvanticity.

The *in vitro* effects of WT and chimeric HLTs on macrophages were especially intriguing. We previously reported that LT-IIc had the exceptional ability to potentiate the release of IL-1α and IL-1β from stimulated macrophages. To our surprise, LT-IIbc that contains the LT-IIc B subunits did not induce a similar reaction in macrophages. Similarly, the chimeric HLT adjuvant LT-IIcb which contains the A subunit of LT-IIc also did not confer a robust IL-1 response from these macrophages (Fig 3). This pattern of responses suggests that neither the A subunit, nor the B subunit of LT-IIc is solely responsible for the potentiation of IL-1. Rather, the effect is due to a unique combination of the LT-IIc holotoxin that affords this functionality. Additionally, we found that WT LT-IIb induced the accumulation of pro-IL-1β within LPS-primed macrophages and that this effect was a unique characteristic of the WT LT-IIb (Fig 3D). The unique properties exhibited by WT LT-IIb and WT LT-IIc were not transferable to either chimeric HLT adjuvants strongly suggested that additional mechanisms are responsible for the actions of these adjuvants, beyond conventional ganglioside binding.

Finally, the requirement for enzymatic activity for adjuvanticity of HLTs has been under considerable debate [11]. To address this question for the enhancement of CD8^+^ T cell responses induced by LT-IIb, we utilized LT-IIb(S59K, E108K) a well described enzymatically deficient mutant [26]. Compared to WT LT-IIb, LT-IIb(S59K, E108K) provided no statistical enhancement to the CD8^+^ T cell response (Fig 6A) nor contributed any improvements to the clearance of rLM-OVA upon challenge one month after vaccination (Fig 6B) when utilized at the 1 μg dose. Unlike previous reports suggesting that an active A subunit may be partially dispensable for humoral adjuvanticity elicited by the HLT adjuvants [27–29], LT-IIb does indeed require a fully active catalytic A polypeptide to fully function as a CD8^+^ T cell adjuvant when delivered by the ID route. However, as seen by the data, there did appear to be a subtle, though not statistically significant, increase in the number of circulating OVA-specific CD8^+^ T cells induced by LT-IIb (S59K, E108K). Thus, it is entirely possible that by substantially escalating the dosage of this enzymatically deficient HLT, a slight CD8+ T cell enhancements would be afforded. However, it is clear that a fully intact A subunit is critical for the robust protection elicited by LT-IIb.

These unique responses elicited by the WT HLT adjuvants discussed thus far are even more complex in the context of Y1 adrenal cell responses (Table 2). It is curious that the chimeric HLT adjuvants, which contained the B subunits from LT-IIb and LT-IIc displayed similar, but not identical, trends of Y1 adrenal cells cAMP responses when compared to the parental WT adjuvant. The Y1 adrenal cells are exquisitely sensitive to cAMP intoxication and has remained the gold standard in the detection and semi- quantification of toxins such as HLTs. However, in light of the data presented, it could be hypothesized that perhaps not all of the experimental responses can be completely attributed to purely cAMP changes. Indeed, the usage of forskolin as a vaccine adjuvants yields no adjuvant response in our experimental models, yet retains an excellent profile of raising cAMP in Y1 adrenal cells (unpublished). Additionally, for example, LT-IIb (T13I), a detoxified mutant induces >1000 fold-less cAMP intoxication as measured by Y1 adrenal cell assay when compared to WT LT-IIb [10, 19]. Yet, LT-IIb (T13I) maintains equivalent adjuvanticity to WT LT-IIb. Thus, cAMP levels, as measured indirectly by Y1 cytotoxic assays, is not predicative of neither the quality nor the magnitude of the adjuvant properties. An alternative explanation could be that the A subunit is actually ribosylation other, non-Gsα targets to facilitate the adjuvant properties. Thus, additional studies to identify potential secondary cellular target of the type II HLT adjuvants utilizing WT and chimeric HLT adjuvants is warranted.

## Conclusion

Effective adjuvants will be a critical component for development of future vaccines as antigen manipulation alone is no longer sufficient to harness the full capacity of the adaptive immune response. Potent vaccine adjuvants that elicit robust and specific immune responses will be critical for the prevention of complex and serious infectious diseases. LT-IIb and LT-IIc enhance both humoral and cellular immunity and, therefore, show great promise as candidates for inclusion in future vaccines.

## Supporting Information

S1 Fig LT-IIb treatment of LPS-stimulated peritoneal macrophages enhances the percentage of the pro-IL-1β+ population. *Statistical Analysis*: One-way ANOVA with Bonferroni post-test. *P ≤ 0.05; **P ≤ 0.01; ***P ≤ 0.001 compared to all other groups unless indicated.
(PDF)
