# Supplementary material for: The Divergent CD8+ T Cell Adjuvant Properties of LT-IIb and LT-IIc, Two Type II Heat-Labile Enterotoxins, Are Conferred by Their Ganglioside-Binding B Subunits
Source: PLoS One. 2015 Nov 13;10(11):e0142942. doi: 10.1371/journal.pone.0142942 (PMC4643920; doi:10.1371/journal.pone.0142942)
Supplement: S1 Fig — Statistical Analysis: One-way ANOVA with Bonferroni post-test. *P ≤ 0.05; **P ≤ 0.01; ***P ≤ 0.001 compared to all other groups unless indicated. (PDF) [file pone.0142942.s001.pdf]

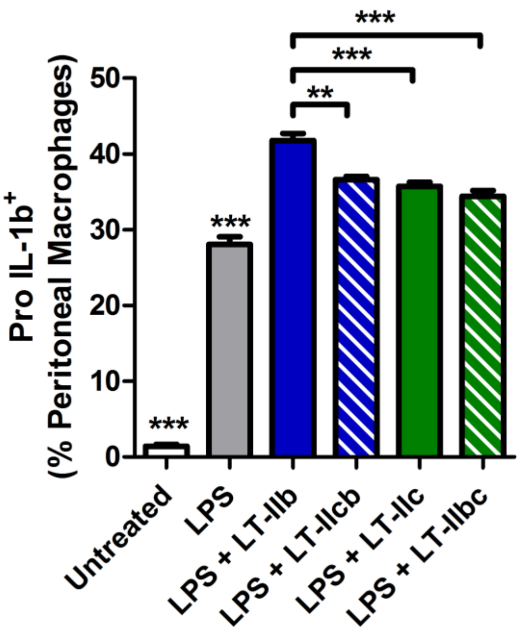

Supporting Figure 1. LT-IIb treatment of LPS-stimulated peritoneal macrophages enhances the percentage of the pro-IL-1β+ population.

Statistical Analysis: One-way ANOVA with Bonferroni post-test. *P ≤ 0.05; **P ≤ 0.01; ***P ≤ 0.001 compared to all other groups unless indicated.
